# PPAR*γ* Attenuates Interleukin-1*β*-Induced Cell Apoptosis by Inhibiting NOX2/ROS/p38MAPK Activation in Osteoarthritis Chondrocytes

**DOI:** 10.1155/2021/5551338

**Published:** 2021-05-04

**Authors:** Su Ni, Dong Li, Hui Wei, Kai-Song Miao, Chao Zhuang

**Affiliations:** ^1^Laboratory of Clinical Orthopedics, The Affiliated Changzhou No.2 People's Hospital of Nanjing Medical University, Changzhou 213003, China; ^2^Department of Orthopedics, The Affiliated Changzhou No.2 People's Hospital of Nanjing Medical University, Changzhou 213003, China

## Abstract

**Introduction:**

Reactive oxygen species (ROS) induced by extracellular cytokines trigger the expression of inflammatory mediators in osteoarthritis (OA) chondrocyte. Peroxisome proliferator-activated receptor gamma (PPAR*γ*) exerts an anti-inflammatory effect. The aim of this study was to elucidate the role of PPAR*γ* in interleukin-1*β*- (IL-1*β*-) induced cyclooxygenase-2 (COX-2) and prostaglandin E_2_ (PGE_2_) expression through ROS generation in OA chondrocytes.

**Methods:**

IL-1*β*-induced ROS generation and chondrocyte apoptosis were determined by flow cytometry. Contents of NADPH oxidase (NOX), caspase-3, and caspase-9 were evaluated by biochemical detection. The involvement of NOX2 and mitogen-activated protein kinases (MAPKs) in IL-1*β*-induced COX-2 and PGE2 expression was investigated using pharmacologic inhibitors and further analyzed by western blotting. Activation of PPAR*γ* was performed by using a pharmacologic agonist and was analyzed by western blotting.

**Results:**

IL-1*β*-induced COX-2 and PGE_2_ expression was mediated through NOX2 activation/ROS production, which could be attenuated by N-acetylcysteine (NAC; a scavenger of ROS), GW1929 (PPAR*γ* agonist), DPI (diphenyleneiodonium chloride, NOX2 inhibitor), SB203580 (p38MAPK inhibitor), PD98059 (extracellular signal-regulated kinase, ERK inhibitor), and SP600125 (c-Jun N-terminal kinase, JNK inhibitor). ROS activated p38MAPK to enter the nucleus, which was attenuated by PPAR*γ*.

**Conclusion:**

In OA chondrocytes, IL-1*β* induced COX-2 and PGE_2_ expression via activation of NOX2, which led to ROS production and MAPK activation. The activation of PPAR*γ* exerted protective roles in the pathogenesis of OA.

## 1. Introduction

Osteoarthritis (OA) is a chronic degenerative arthritis. The clinical manifestations are joint pain, stiffness, dysfunction, and deformity, which often seriously affect the life of patients [1, 2]. Its incidence increases with age, which has become the main cause of disability of the elderly. The potential initiation of OA enhanced chondrocyte apoptosis [3]. The apoptosis rate of normal articular chondrocytes is very low (2%-5%), while the apoptosis rate of OA articular chondrocytes is significantly increased from 18% to 21%, indicating that chondrocyte apoptosis is involved in OA. This is often accompanied by abnormal signal transduction between chondrocytes, suggesting that intercellular signal transduction plays an important role in the occurrence and development of OA [4].

Peroxisome proliferator-activated receptor gamma (PPAR*γ*) is a ligand-induced transcription factor known to play a role in normal cell function. After activation, PPAR*γ* binds to a specific response element (PPRE) with retinol X receptor (RXR) heterodimer and promotes the expression of the target gene [5]. The PPAR*γ* level is elevated during preadipocyte differentiation and plays a central role in lipid metabolism, glucose homeostasis, inflammation, and cell proliferation [6, 7]. A recent study has shown that PPAR*γ* is a key regulator of cartilage health, and the lack of PPAR*γ* leads to the acceleration of spontaneous OA [8]. Activation of PPAR*γ* is thought to have protective effects on cell apoptosis and inflammation in OA.

NADPH oxidase (NOX) is an enzyme complex composed of membrane subunits gp91^phox^ (NOX2) and p22^phox^, cytoplasmic subunits p47^phox^, p67^phox^, and p40^phox^, and small molecule GTPase-binding protein Rac. Its catalytic subunit gp91^phox^ and its homologues NOX1, NOX3, NOX4, NOX5, DUOX1, and DUOX2 are known as the NOX family, which exists in almost all organs, tissues, and cells [9]. The main biological function of NOX family proteins is to produce reactive oxygen species (ROS), which can maintain the normal physiological activities of cells. However, under abnormal conditions, such as the stimulation of growth factors, cytokines, and inflammatory mediators, the elevated NOX proteins produce excessive ROS [10].

ROS is considered to be an important homeostatic factor in the development of OA. ROS produced by mitochondrial electron transfer is beneficial to maintain the redox balance of glycolysis, so as to maintain the dynamic balance of metabolism of articular chondrocytes [11]. ROS has immune and signal transduction functions under a steady state, but ROS imbalance can cause a variety of pathological changes [12].

Mitogen-activated protein kinases (MAPKs) are a class of serine/threonine protein kinases widely existing in mammals. They exist in most cells and are important transmitters of extracellular signal transduction to the nucleus [13]. MAPKs participate in and regulate the proliferation, differentiation, and apoptosis of chondrocytes in articular cartilage, and the imbalance of MAPKs plays a very important role in the occurrence and development of OA [14].

In this study, we used IL-1*β* to induce OA in vitro. We found that PPAR*γ* activity decreased and chondrocyte apoptosis increased. We inferred that increasing PPAR*γ* activity could reduce the production of cyclooxygenase-2 (COX-2) and prostaglandin E_2_ (PGE_2_) through the NOX2/ROS/p38MAPK signal axis, thus reducing chondrocyte apoptosis and alleviating OA progress.

## 2. Materials and Methods

### 2.1. Reagents

Collagenase II was obtained from Sigma (St. Louis, MO, USA). Dulbecco's modified Eagle's medium (DMEM) supplied with 100 U penicillin and 100 *μ*g streptomycin and 10% fetal bovine serum (FBS) was from Gibco (Grand Island, NY, USA). Cell Counting Kit-8 (CCK-8), FITC Annexin V Apoptosis Detection Kit, 0.25% trypsin, ROS assay kit, PGE_2_ ELISA kit, NOX, caspase-9 and caspase-3 colorimetric assay kits, and BCA protein assay kit were purchased from Beyotime Biotechnology (Shanghai, China). TRIzol was from Invitrogen (Carlsbad, CA, USA). The High-Capacity cDNA Reverse Transcription kit was obtained from Applied Biosystems (Foster City, CA, USA). SYBR Select Master Mix was obtained from Applied Biosystems (Austin, TX, USA). Collagenase II was dissolved to 2 mg/ml in DMEM to digest articular cartilage. N-acetylcysteine (NAC), PPAR*γ* agonist (GW1929), NOX2 inhibitor (DPI), p38MAPK inhibitor (SB203580), ERK inhibitor (PD98059), and JNK inhibitor (SP600125) were purchased from Cell Signaling Technology (Danvers, MA, USA) and dissolved in dimethyl sulfoxide (DMSO).

### 2.2. Rat Chondrocyte Isolation and Culture

The rat chondrocytes were isolated and cultured as described previously [15]. In brief, chondrocytes were isolated from the articular cartilages of four-week-old male Sprague-Dawley rats. The cartilages were removed from animals that were subsequently euthanized via an overdose of anesthesia. The cartilages were cut into thin slices, washed with sterile phosphate-buffered saline (PBS), and then digested with 1 mg/ml collagenase type II in DMEM for 5 h at 37°C within an incubator. The digested cartilages were collected and centrifuged. The pellets were resuspended in DMEM and filtered through a 70 *μ*m nylon cell strainer (FALCON, Pittsburgh, PA, USA). The primary chondrocytes were cultured in DMEM supplemented with 10% FBS, 100 U penicillin, and 100 *μ*g streptomycin in a 5% CO_2_ incubator at 37°C. Confluent chondrocytes were split in 1 : 2 ratios up to passages 2-3 and used for subsequent experiments. This study was reviewed and approved by the ethics committee of the No.2 People's Hospital of Changzhou, Jiangsu, China.

### 2.3. Cell Immunofluorescence Assay

Cultured chondrocytes were seeded on the gelatin precoated slices and grew to 50%-60% confluence. Then, the slices were fixed with 4% formaldehyde for 30 min, penetrated with 0.5% Triton X-100 for 10 min, and blocked with 1% BSA for 1 h at room temperature. Rabbit anti-rat collagen II antibody and rabbit anti-rat SOX9 antibody (antibodies were purchased from Abcam and diluted in 1 : 1000) were added and incubated in a wet box at 4°C overnight. The fluorescent second antibody was added, incubated in a wet box, and placed at room temperature for 1 h. Antiquenching tablets and DAPI 1 : 500 diluted slices were stored in a refrigerator at -20°C and photographed under a fluorescence microscope (Eclipse Ni, NIKON, Japan).

### 2.4. Cell Viability Assay

To assess the time and concentration-response relationship of IL-1*β* in the experiment, the cell viability was evaluated by the CCK-8 assay. Chondrocytes were plated in 96-well plates at a density of 5 × 10^3^ cells/well to adhere overnight and treated with 0 ng/ml, 1 ng/ml, 10 ng/ml, or 30 ng/ml IL-1*β* for 0 min, 10 min, 30 min, 1 h, 2 h, 12 h, 24 h, and 48 h. After incubation time, 10 *μ*l of CCK-8 solution was added to each well and further incubated for 1 h at 37°C in 5% CO_2_. Absorbance at 450 nm was measured using a microplate reader (SpectraMax Plus 384, MD, USA).

### 2.5. Cell Treatment

Cultured chondrocytes were pretreated with 10 mM NAC, 10 *μ*M GW1929, 10 *μ*M DPI, 10 *μ*M SB203580, 10 *μ*M PD98059, or 10 *μ*M SP600125 for 1 h and then treated with 10 ng/ml IL-1*β*, cell proliferation was detected by the CCK-8 assay (0 h, 12 h, 24 h, and 48 h), cell apoptosis (24 h) and ROS level (24 h) were detected by flow cytometry, PGE_2_ content in cell supernatant was detected by ELISA (24 h), and COX-2 expression was detected by qRT-PCR (24 h). Cultured chondrocytes were pretreated with 10 mM NAC, 10 *μ*M GW1929, or 10 *μ*M DPI for 1 h and then treated with 10 ng/ml IL-1*β*; western blot was used to detect the expression of PPAR*γ*, NOX2, p38, p-p38, ERK1/2, p-ERK1/2, JNK1/2, and p-JNK1/2 (24 h).

### 2.6. Cell Apoptosis Detection

To quantify the percentage of chondrocytes undergoing apoptosis, the FITC Annexin V Apoptosis Detection Kit was used as described previously [16]. Briefly, after treatment, chondrocytes were harvested and washed twice with cold PBS, then resuspended in 100 *μ*l binding buffer into which 5 *μ*l of FITC Annexin V and 5 *μ*l propidium iodide (PI) were added for 15 min at 25°C in the dark. After incubation, additional 400 *μ*l binding buffer was added, and chondrocytes were analyzed with a FACScan flow cytometer (BD Biosciences, San Jose, CA, USA). Results were quantified and assembled by the FlowJo software (Tree Star, Inc., USA).

### 2.7. The Measurement of ROS Level

The ROS level in chondrocytes was measured by using a commercialized kit according to the manufacturer's optimized instructions. Briefly, the number of chondrocytes to be tested was counted using a cell count board. Then, cells were suspended in diluted DCFH-DA (10 mM) in PBS and incubated at 37°C for 20 min. After washing twice with PBS, ROS production in these cells was measured by using a FACScan flow cytometer (BD Biosciences, San Jose, CA, USA).

### 2.8. Determination of PGE_2_

The PGE_2_ in cell supernatant was measured by ELISA according to the manufacturer's protocol. Optical density was detected by the microplate reader at 450 nm (SpectraMax Plus 384, MD, USA).

### 2.9. Quantitative Real-Time Reverse Transcription Polymerase Chain Reaction (qRT-PCR)

Total RNA was extracted from treated samples using TRIzol. The High-Capacity cDNA Reverse Transcription kit was used to reverse transcribe total RNA (1 *μ*g) according to the manufacturer's protocol. COX-2 and glyceraldehyde 3-phosphate dehydrogenase (GAPDH) were amplified using SYBR® Select Master Mix in a Bio-Rad iQ5 real-time PCR system. The specific primer sequences (designed by Sangon Biotech. Co., Ltd, Shanghai, China) are presented in Table 1. The relative expression levels were calculated by the comparative threshold cycle method.

### 2.10. Determination of NADPH Oxidase Activity

Cells were grown in six-well culture plates and incubated with endosulfan for the indicated time intervals. Cells were gently scraped and centrifuged for 10 min at 4°C. The cell pellet was resuspended in 35 ml of ice-cold DMEM medium per vial and then kept on ice. 5 *μ*l of the cell suspension was added to a final volume of 200 ml of prewarmed DMEM medium containing either NADPH (1 *μ*M) or lucigenin (20 *μ*M), to initiate the reaction, followed by immediate measurement of chemiluminescence using a luminometer (Appliskan, Thermo, USA).

### 2.11. Measurement of Caspase-9 and Caspase-3 Activities

Caspase-9 and caspase-3 activities were measured by colorimetric assay kits according to the manufacturer's instructions. Briefly, cells were collected and lysed using the lysis buffer provided. The caspase-9 and caspase-3 activity colorimetric assays are based on the hydrolysis of the peptide substrate acetyl, resulting in the release of p-nitroaniline moiety, which has a high absorbance at 405 nm that was detected by a microplate reader (SpectraMax Plus 384, MD, USA).

### 2.12. Western Blot

Chondrocytes were harvested and lysed in RIPA buffer for total protein extraction. The protein concentration of each sample was determined by the BCA protein assay kit. After that, 10 *μ*g of protein was separated by electrophoresis on 10% sodium dodecyl sulfate-polyacrylamide gels and transferred to a polyvinylidene fluoride (PVDF) membrane. After being blocked for 1 h at room temperature in tris buffered saline with Tween-20 with 5% nonfat milk, the PVDF membrane was then incubated with rabbit anti-rat PPAR*γ* antibody, rabbit anti-rat NOX2 antibody, rabbit anti-rat p38 antibody, rabbit anti-rat p-p38 antibody, rabbit anti-rat ERK1/2 antibody, rabbit anti-rat p-ERK1/2 antibody, rabbit anti-rat JNK1/2 antibody, rabbit anti-rat p-JNK1/2 antibody, and rabbit anti-rat GAPDH antibody, respectively (all these antibodies were purchased from Cell Signaling Technology and diluted in 1 : 1000) overnight at 4°C and then with horseradish peroxidase-conjugated secondary antibodies for 1 h at room temperature. The blots were detected with the enhanced chemiluminescence assay kit. The GAPDH signal was used as an internal loading control, and relative expression levels were quantified by Quantity One software (Bio-Rad Laboratories, Hercules, CA, USA).

### 2.13. Statistical Analysis

Data shown in our study were represented as means ± SD from three independent experiments. One-way ANOVA was conducted for comparison between multiple groups, and *P* < 0.05 was considered to be statistically significant.

## 3. Results

### 3.1. Identification of Primary Cultured Rat Chondrocytes

**Figure 1:**
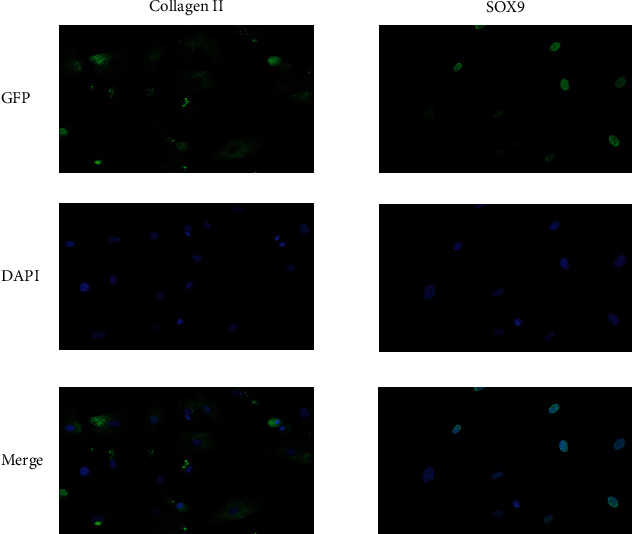
Cell immunofluorescence. Cultured cells were identified as chondrocytes by immunofluorescence staining of collagen II and SOX9.

The primary cultured cells were identified as chondrocytes by immunofluorescence staining of collagen II and SOX9 (Figure 1).

### 3.2. High Level of IL-1*β* Impaired the Viability of Chondrocytes

**Figure 2:**
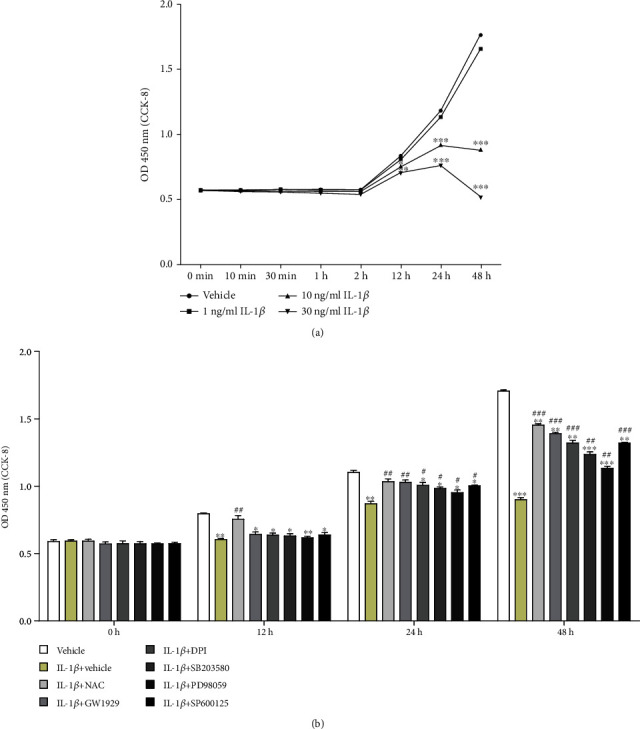
Cell viability. (a) Cultured chondrocytes were treated with 0 ng/ml, 1 ng/ml, 10 ng/ml, or 30 ng/ml IL-1*β* for 0 min, 10 min, 30 min, 1 h, 2 h, 12 h, 24 h, and 48 h, respectively. Cell viability was detected by CCK-8. (b) Cultured chondrocytes were pretreated with 10 mM NAC, 10 *μ*M GW1929, 10 *μ*M DPI, 10 *μ*M SB203580, 10 *μ*M PD98059, or 10 *μ*M SP600125 for 1 h and then treated with 10 ng/ml IL-1*β* for 0 h, 12 h, 24 h, and 48 h. Cell viability was detected by CCK-8. Results are presented as means ± standard deviation of three independent experiments. Chondrocytes cultured in DMEM were used as the vehicle control. ^∗^*P* < 0.05, ^∗∗^*P* < 0.01, and ^∗∗∗^*P* < 0.001 versus the vehicle control. ^#^*P* < 0.05, ^##^*P* < 0.01, and ^###^*P* < 0.001 versus IL-1*β* group.

Cell viability was measured at different time points (0 min, 10 min, 30 min, 1 h, 2 h, 12 h, 24 h, and 48 h) after different concentrations of IL-1*β* continuous treatments (0 ng/ml, 1 ng/ml, 10 ng/ml, and 30 ng/ml). The results showed that the viability of chondrocytes decreased by 0.12% (*P* > 0.05), 0.35% (*P* > 0.05), 0.52% (*P* > 0.05), 1.33% (*P* > 0.05), 2.87% (*P* > 0.05), 4.14% (*P* > 0.05), and 6.04% (*P* > 0.05) after 1 ng/ml IL-1*β* treatment at seven time points compared with the vehicle group, respectively. After 10 ng/ml IL-1*β* treatment, the viability of chondrocytes decreased by 0.58% (*P* > 0.05), 1.79% (*P* > 0.05), 2.48% (*P* > 0.05), 3.18% (*P* > 0.05), 9.72% (*P* < 0.05), 22.31% (*P* < 0.001), and 6.04% (*P* < 0.001) at seven time points compared with the vehicle group, respectively. After 30 ng/ml IL-1*β* treatment, the viability of chondrocytes decreased by 1.74% (*P* > 0.05), 3.18% (*P* > 0.05), 4.90% (*P* > 0.05), 6.24% (*P* > 0.05), 15.86% (*P* < 0.01), 35.62% (*P* < 0.001), and 70.74% (*P* < 0.001) at seven time points compared with the vehicle group, respectively (Figure 2(a)). Since the 0 ng/ml group showed no change and few cells survived in the 30 ng/ml group, we eventually chose 10 ng/ml IL-1*β* for further intervention. Cultured chondrocytes were pretreated with 10 mM NAC, 10 *μ*M GW1929 (PPAR*γ* agonist), 10 *μ*M DPI (NOX2 inhibitor), 10 *μ*M SB203580 (p38MAPK inhibitor), 10 *μ*M PD98059 (ERK inhibitor), or10 *μ*M SP600125 (JNK inhibitor), respectively, for 1 h and then treated with 10 ng/ml IL-1*β*. Cell proliferation was detected at 12 h, 24 h, and 48 h. Compared with the vehicle group, the viability of chondrocytes decreased by 7.97% (IL-1*β* group, *P* < 0.01), 2.22% (NAC group, *P* > 0.05), 3.51% (GW1929 group, *P* < 0.05), 4.41% (DPI group, *P* < 0.05), 5.17% (SB203580 group, *P* < 0.05), 7.32% (PD98059 group, *P* < 0.01), and 4.11% (SP600125 group, *P* < 0.05) at 12 h, respectively. There was a significant difference between the IL-1*β* group and the NAC group (*P* < 0.01). There was no significant difference between the IL-1*β* group and agonist or inhibitor groups (*P* > 0.05). Compared with the vehicle group, the viability of chondrocytes decreased by 21.34% (IL-1*β* group, *P* < 0.01), 6.20% (NAC group, *P* > 0.05), 7.83% (GW1929 group, *P* > 0.05), 9.86% (DPI group, *P* < 0.05), 11.89% (SB203580 group, *P* < 0.05), 14.64% (PD98059 group, *P* < 0.01), and 10.22% (SP600125 group, *P* < 0.05) at 24 h, respectively. There were significant differences between the IL-1*β* group and NAC or agonist or inhibitor groups. Compared with the vehicle group, the viability of chondrocytes decreased by 45.75% (IL-1*β* group, *P* < 0.001), 14.85% (NAC group, *P* < 0.01), 17.90% (GW1929 group, *P* < 0.01), 22.09% (DPI group, *P* < 0.01), 27.07% (SB203580 group, *P* < 0.001), 33.15% (PD98059 group, *P* < 0.001), and 22.07% (SP600125 group, *P* < 0.01) at 48 h, respectively. There were significant differences between the IL-1*β* group and the NAC or agonist or inhibitor groups (Figure 2(b)).

### 3.3. IL-1*β* Significantly Increased Chondrocyte Apoptosis

**Figure 3:**
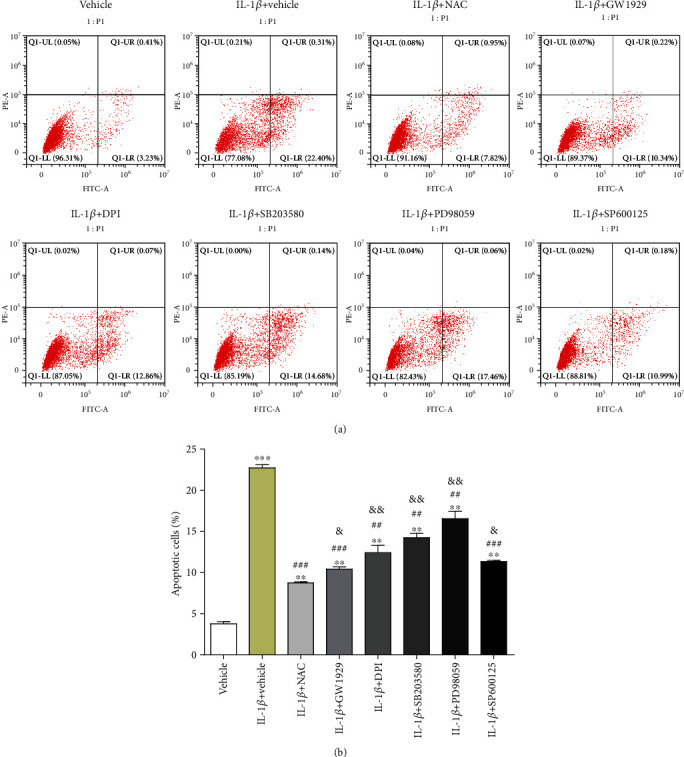
Cell apoptosis. (a) Cultured chondrocytes were pretreated with 10 mM NAC, 10 *μ*M GW1929, 10 *μ*M DPI, 10 *μ*M SB203580, 10 *μ*M PD98059, or 10 *μ*M SP600125 for 1 h and then treated with 10 ng/ml IL-1*β* for 24 h. Chondrocytes cultured in DMEM were used as the vehicle control. FITC annexin V/PI staining and flow cytometry assays were used to detect cell apoptosis. (b) Quantification of apoptosis in different groups. Results are presented as means ± standard deviation of three independent experiments. ^∗∗^*P* < 0.01 and ^∗∗∗^*P* < 0.001 versus the vehicle control. ^##^*P* < 0.01 and ^###^*P* < 0.001 versus the IL-1*β* group. ^&^*P* < 0.05 and ^&&^*P* < 0.01 versus the NAC group.

The chondrocytes were treated as described above, and the apoptosis ratio was detected by the flow cytometry assay (Figure 3(a)). Chondrocytes cultured in DMEM were used as a vehicle control. The percentage of apoptotic cells in quadrants Q1-UR and Q1-LR was calculated and is shown in Figure 3(b). When treated with IL-1*β*, the apoptotic rate of chondrocytes increased by about 5 times (*P* < 0.001) compared with the vehicle group. When pretreated with NAC or agonist or inhibitors, the apoptotic rate increased by 1.31 times (NAC group, *P* < 0.01), 1.74 times (GW1929 group, *P* < 0.01), 2.27 times (DPI group, *P* < 0.01), 2.76 times (SB203580 group, *P* < 0.01), 3.36 times (PD98059 group, *P* < 0.01), or 2 times (SP600125 group, *P* < 0.01), respectively, compared with the vehicle group. Among all these chemicals, NAC showed the best antiapoptotic effect, decreased by 61.46% (*P* < 0.001), compared with the IL-1*β* group. Meanwhile, the other compounds all possessed different degrees of antiapoptosis, which presented as 54.20% (GW1929 group, *P* < 0.001), 45.44% (DPI group, *P* < 0.01), 37.30% (SB203580 group, *P* < 0.01), 27.18% (PD98059 group, *P* < 0.01), and 50.10% (SP600125 group, *P* < 0.001) compared with the IL-1*β* group, respectively. Compared with the NAC group, the apoptotic rate increased by 18.83% (GW1929 group, *P* < 0.05), 41.55% (DPI group, *P* < 0.01), 62.67% (SB203580 group, *P* < 0.01), 88.92% (PD98059 group, *P* < 0.01), or 29.45% (SP600125 group, *P* < 0.05), respectively (Figure 3(b)).

### 3.4. The Elevated Oxidative Stress and Inflammation Factors Induced by IL-1*β* Can Be Partially Reversed by NAC, PPAR*γ* Agonist, and MAPK Inhibitors

**Figure 4:**
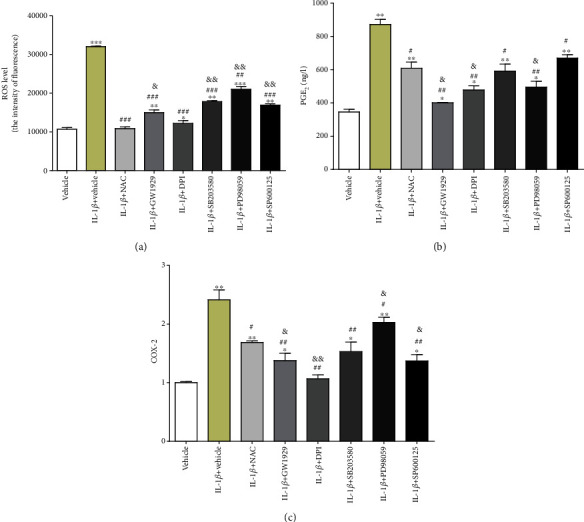
Detection of oxidative stress and inflammatory factors. Cultured chondrocytes were pretreated with 10 mM NAC, 10 *μ*M GW1929, 10 *μ*M DPI, 10 *μ*M SB203580, 10 *μ*M PD98059, or 10 *μ*M SP600125 for 1 h and then treated with 10 ng/ml IL-1*β* for 24 h. (a) ROS level was detected by flow cytometry, (b) PGE_2_ content was detected by ELISA, and (c) COX-2 expression was detected by qRT-PCR. Results are presented as means ± standard deviation of three independent experiments. Chondrocytes cultured in DMEM were used as the vehicle control. ^∗^*P* < 0.05, ^∗∗^*P* < 0.01, and ^∗∗∗^*P* < 0.001 versus the vehicle control. ^#^*P* < 0.05, ^##^*P* < 0.01, and ^###^*P* < 0.001 versus the IL-1*β* group. ^&^*P* < 0.05 and ^&&^*P* < 0.01 versus the NAC group.

The level of intracellular ROS in chondrocytes was considered an indicator of oxidative stress. With the presence of IL-1*β*, the fluorescence intensity of intracellular ROS was 2 times higher than that of the vehicle group (*P* < 0.001), which indicated enhanced intracellular oxidative stress. When pretreated with the chemicals, the fluorescence intensity of intracellular ROS increased by 9% (NAC group, *P* > 0.05), 40.18% (GW1929 group, *P* < 0.01), 14.01% (DPI group, *P* < 0.05), 67.28% (SB203580 group, *P* < 0.01), 96.26% (PD98059 group, *P* < 0.001), or 57.94% (SP600125 group, *P* < 0.01), respectively, compared with the vehicle group, but decreased by 65.93% (NAC group, *P* < 0.001), 53.12% (GW1929 group, *P* < 0.001), 61.87% (DPI group, *P* < 0.001), 44.06% (SB203580 group, *P* < 0.001), 34.37% (PD98059 group, *P* < 0.01), or 47.18% (SP600125 group, *P* < 0.001), respectively, compared with the IL-1*β* group. NAC showed the best antioxidative effect according to our data. Compared with the NAC group, the fluorescence intensity of intracellular ROS increased by 37.61% (GW1929 group, *P* < 0.05), 11.92% (DPI group, *P* > 0.05), 64.22% (SB203580 group, *P* < 0.01), 92.66% (PD98059 group, *P* < 0.01), or 55.04% (SP600125 group, *P* < 0.01), respectively (Figure 4(a)).

PGE_2_ is a common inflammatory cytokine in OA, which can be induced by various stimuli including IL-1*β*. After the application of IL-1*β*, PGE_2_ content in cell supernatant increased by 1.51 times than that of the vehicle group (*P* < 0.01). When pretreated with NAC or agonist or inhibitors, PGE_2_ content in cell supernatant increased by 75.72% (NAC group, *P* < 0.01), 16.18% (GW1929 group, *P* < 0.05), 38.15% (DPI group, *P* < 0.05), 70.80% (SB203580 group, *P* < 0.01), 43.35% (PD98059 group, *P* < 0.05), or 93.64% (SP600125 group, *P* < 0.01), respectively, compared with the vehicle group, but decreased by 43.42% (NAC group, *P* < 0.05), 116% (GW1929 group, *P* < 0.01), 82.42% (DPI group, *P* < 0.01), 47.54% (SB203580 group, *P* < 0.05), 75.80% (PD98059 group, *P* < 0.01), or 30.14% (SP600125 group, *P* < 0.05), respectively, compared with the IL-1*β* group. Compared with the NAC group, PGE_2_ content in cell supernatant decreased by 51.24% (GW1929 group, *P* < 0.05), 27.19% (DPI group, *P* < 0.05), 2.87% (SB203580 group, *P* > 0.05), or 22.58% (PD98059 group, *P* < 0.05) or increased by 10.19% (SP600125 group, *P* > 0.05), respectively (Figure 4(b)).

The mRNA expression of COX-2 was upregulated (increased by 1.42 times, *P* < 0.01) in chondrocytes treated with IL-1*β*, compared with the vehicle group. When pretreated with NAC or agonist or inhibitors, the mRNA expression of COX-2 increased by 69% (NAC group, *P* < 0.01), 38% (GW1929 group, *P* < 0.05), 7% (DPI group, *P* > 0.05), 53% (SB203580 group, *P* < 0.05), 103% (PD98059 group, *P* < 0.01), or 37% (SP600125 group, *P* < 0.05), respectively, compared with the vehicle group, but decreased by 43.19% (NAC group, *P* < 0.05), 75.36% (GW1929 group, *P* < 0.01), 126% (DPI group, *P* < 0.01), 58.16% (SB203580 group, *P* < 0.05), 19.21% (PD98059 group, *P* < 0.05), or 76.64% (SP600125 group, *P* < 0.01), respectively, compared with the IL-1*β* group. Compared with the NAC group, the mRNA expression of COX-2 decreased by 22.46% (GW1929 group, *P* < 0.05), 57.94% (DPI group, *P* < 0.01), 10.45% (SB203580 group, *P* > 0.05), or 23.35% (SP600125 group, *P* < 0.05) or increased by 20.11% (PD98059 group, *P* < 0.05), respectively (Figure 4(c)).

### 3.5. NOX and Caspase Activities in IL-1*β*-Induced Chondrocyte Apoptosis

**Figure 5:**
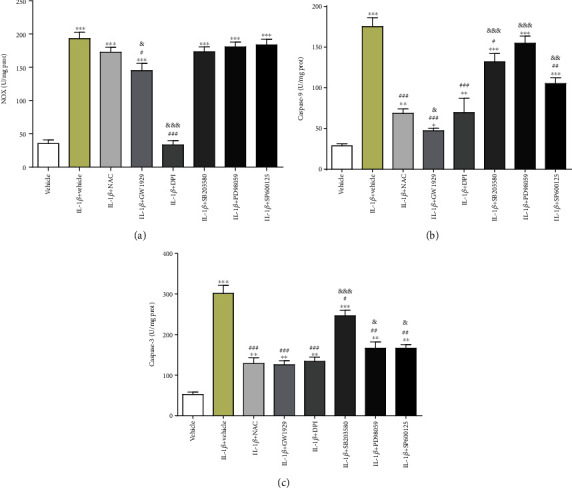
The measurement of NOX and caspase activities. Cultured chondrocytes were pretreated with 10 mM NAC, 10 *μ*M GW1929, 10 *μ*M DPI, 10 *μ*M SB203580, 10 *μ*M PD98059, or 10 *μ*M SP600125 for 1 h and then treated with 10 ng/ml IL-1*β* for 24 h. Chondrocytes were harvested, and total proteins were extracted, and the levels of NOX and cleaved caspases were detected by the assay kit: (a) NOX, (b) caspase-9, and (c) caspase-3. Results are presented as means ± standard deviation of three independent experiments. Chondrocytes cultured in DMEM were used as the vehicle control. ^∗^*P* < 0.05, ^∗∗^*P* < 0.01, and ^∗∗∗^*P* < 0.001 versus the vehicle control. ^#^*P* < 0.05, ^##^*P* < 0.01, and ^###^*P* < 0.001 versus the IL-1*β* group. ^&^*P* < 0.05, ^&&^*P* < 0.01, and ^&&&^*P* < 0.001 versus the NAC group.

With the presence of IL-1*β*, the NOX activity increased by 5.5 times than the vehicle group (*P* < 0.001). When pretreated with other chemicals, the levels of NOX increased by 4.9 times (NAC group, *P* < 0.001), 4.1 times (GW1929 group, *P* < 0.001), 4.9 times (SB203580 group, *P* < 0.001), 5.1 times (PD98059 group, *P* < 0.001), or 5.2 times (SP600125 group, *P* < 0.001), respectively, but decreased by 6% (DPI group, *P* > 0.05) compared with the vehicle group. The NOX activities decreased by 11% (NAC group, *P* > 0.05), 25% (GW1929 group, *P* < 0.05), 83% (DPI group, *P* < 0.001), 11% (SB203580 group, *P* > 0.05), 7% (PD98059 group, *P* > 0.05), or 6% (SP600125 group, *P* > 0.05), respectively, compared with the IL-1*β* group. Compared with the NAC group, the levels of NOX decreased by 16% (GW1929 group, *P* < 0.05) or 81% (DPI group, *P* < 0.001) but increased by 1% (SB203580 group, *P* > 0.05), 5% (PD98059 group, *P* > 0.05), or 6% (SP600125 group, *P* > 0.05), respectively (Figure 5(a)).

After treatment with IL-1*β*, the level of caspase-9 increased by 5.8 times than the vehicle group (*P* < 0.001). When pretreated with NAC or agonist or inhibitors, the caspase-9 activities increased by 2.4 times (NAC group, *P* < 0.01), 1.6 times (GW1929 group, *P* < 0.05), 2.5 times (DPI group, *P* < 0.01), 4.7 times (SB203580 group, *P* < 0.001), 5.1 times (PD98059 group, *P* < 0.001), or 3.7 times (SP600125 group, *P* < 0.001), respectively, compared with the vehicle group. The levels of caspase-9 decreased by 62% (NAC group, *P* < 0.001), 74% (GW1929 group, *P* < 0.001), 60% (DPI group, *P* < 0.001), 25% (SB203580 group, *P* < 0.05), 12% (PD98059 group, *P* > 0.05), or 40% (SP600125 group, *P* < 0.01), respectively, compared with the IL-1*β* group. Compared with the NAC group, the caspase-9 activities decreased by 31% (GW1929 group, *P* < 0.05) but increased by 2% (DPI group, *P* > 0.05), 94% (SB203580 group, *P* < 0.001), 120% (PD98059 group, *P* < 0.001), or 54% (SP600125 group, *P* < 0.01), respectively (Figure 5(b)).

With the presence of IL-1*β*, the level of caspase-3 increased by 5.6 times than the vehicle group (*P* < 0.001). When pretreated with NAC or agonist or inhibitors, the caspase-3 activities increased by 2.5 times (NAC group, *P* < 0.01), 2.4 times (GW1929 group, *P* < 0.01), 2.4 times (DPI group, *P* < 0.01), 4.6 times (SB203580 group, *P* < 0.001), 3.2 times (PD98059 group, *P* < 0.01), or 3.1 times (SP600125 group, *P* < 0.01), respectively, compared with the vehicle group. The levels of caspase-3 decreased by 58% (NAC group, *P* < 0.001), 59% (GW1929 group, *P* < 0.001), 56% (DPI group, *P* < 0.001), 19% (SB203580 group, *P* < 0.05), 45% (PD98059 group, *P* < 0.01), or 46% (SP600125 group, *P* < 0.01), respectively, compared with the IL-1*β* group. Compared with the NAC group, the caspase-3 activities decreased by 3% (GW1929 group, *P* > 0.05) but increased by 4% (DPI group, *P* > 0.05), 92% (SB203580 group, *P* < 0.001), 29% (PD98059 group, *P* < 0.05), or 30% (SP600125 group, *P* < 0.05), respectively (Figure 5(c)).

### 3.6. IL-1*β* Induced MAPK Signaling Activation and PPAR*γ* Inhibition Could Be Abolished by Different Chemicals

**Figure 6:**
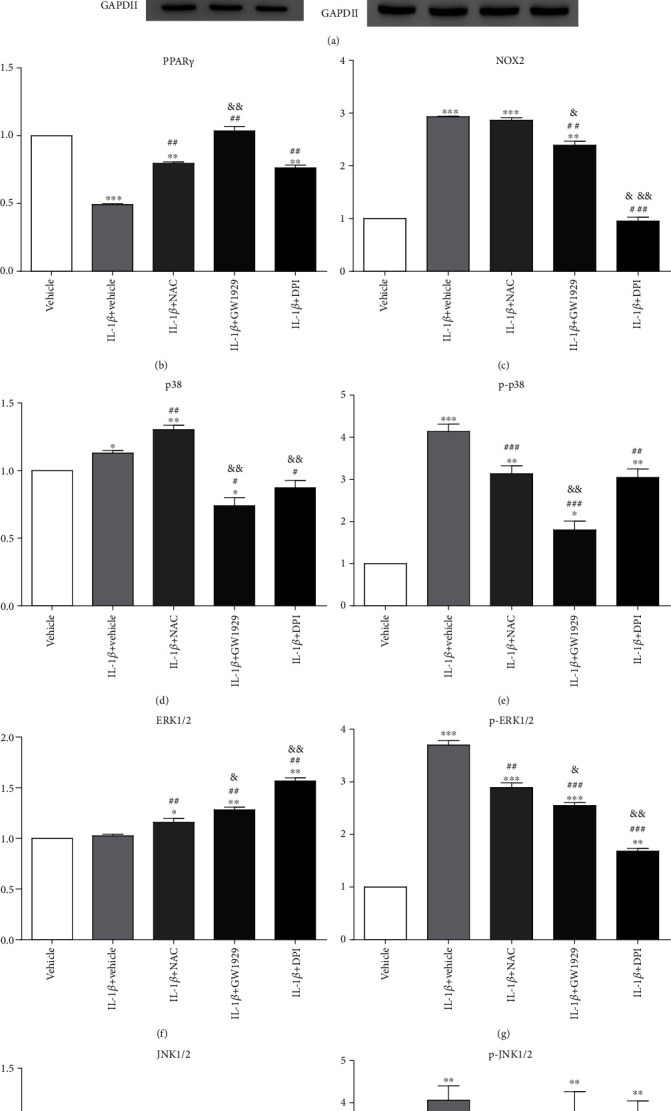
Western blot. Cultured chondrocytes were pretreated with 10 mM NAC, 10 *μ*M GW1929, or 10 *μ*M DPI for 1 h and then treated with 10 ng/ml IL-1*β* for 24 h; western blot was used to detect the expression of PPAR*γ*, NOX2, p38, p-p38, ERK1/2, p-ERK1/2, JNK1/2, and p-JNK1/2. Representative western blot (a) and quantification data (b–i) are shown, respectively. The relative protein levels were normalized to the level of the internal control, GAPDH, and presented as fold changes relative to the control group (the level of the control group was set as 1). Results are presented as the mean ± standard deviation of three independent experiments. Chondrocytes cultured in DMEM were used as the vehicle control. ^∗^*P* < 0.05, ^∗∗^*P* < 0.01, and ^∗∗∗^*P* < 0.001 versus the vehicle control. ^#^*P* < 0.05, ^##^*P* < 0.01, and ^###^*P* < 0.001 versus the IL-1*β* group. ^&^*P* < 0.05 and ^&&^*P* < 0.01 versus the NAC group.

After treatment with IL-1*β*, the activation of PPAR*γ* decreased by 50% compared with the vehicle group (*P* < 0.001). Pretreated with GW1929, PPAR*γ* was activated, and there was no significant difference between the vehicle group and the GW1929 group (*P* > 0.05). The effect of GW1929 was better than that of the NAC group (PPAR*γ* activity increased by 20%, *P* < 0.01). Pretreated with DPI, PPAR*γ* activity was lower than that of the vehicle group (decreased by 24%, *P* < 0.01), but higher than that of the IL-1*β* group (increased by 26%, *P* < 0.01), and there was no significant difference between the DPI group and the NAC group (*P* > 0.05) (Figure 6(b)).

After treatment with IL-1*β*, the activation of NOX2 increased by 200% compared with the vehicle group (*P* < 0.001). There was no significant difference between the IL-1*β* group and the NAC group (*P* > 0.05). Pretreated with GW1929, NOX2 activity increased by 140% compared with the vehicle group (*P* < 0.01), but decreased by 20% compared with the IL-1*β* group (*P* < 0.01). The effect of GW1929 was weaker than that of the NAC group (NOX2 activity decreased by 20.08%, *P* < 0.05). Pretreated with DPI, NOX2 activity returned to normal and there was no significant difference between the vehicle group and the DPI group (*P* > 0.05) (Figure 6(c)).

After treatment with IL-1*β*, p38 activity increased by 13% (*P* < 0.05), while p-p38 activity increased by 300% (*P* < 0.001), compared with the vehicle group. Pretreated with GW1929, the activation of p38 decreased by 26% compared with the vehicle group (*P* < 0.05) and decreased by 34% compared with the IL-1*β* group (*P* < 0.01), while p-p38 activity increased by 80% compared with the vehicle group (*P* < 0.05) but decreased by 56% compared with the IL-1*β* group (*P* < 0.001). Both p38 and p-p38 activities were weaker than that of the NAC group (*P* < 0.01). Pretreated with DPI, the activation of p38 decreased by 38% compared with the IL-1*β* group (*P* < 0.05) and 49.42% compared with the NAC group (*P* < 0.01). There was no significant difference between the vehicle group and the DPI group (*P* > 0.05), while p-p38 activity increased by 200% compared with the vehicle group (*P* < 0.01) but decreased by 26% compared with the IL-1*β* group (*P* < 0.01). There was no significant difference between the NAC group and the DPI group (*P* > 0.05) (Figures 6(d) and 6(e)).

After treatment with IL-1*β*, there was no significant difference in ERK1/2 activity between the vehicle group and the IL-1*β* group (*P* > 0.05), but p-ERK1/2 activity increased by 270% compared with the vehicle group (*P* < 0.001). Pretreated with GW1929, the activation of ERK1/2 increased by 28% compared with the vehicle group (*P* < 0.01) and increased by 20% compared with the IL-1*β* group (*P* < 0.01). ERK1/2 activity was stronger than that of the NAC group (*P* < 0.05), while p-ERK1/2 activity increased by 150% compared with the vehicle group (*P* < 0.001) but decreased by 30% compared with the IL-1*β* group (*P* < 0.01). The activation of p-ERK1/2 was weaker than that of the NAC group (*P* < 0.05). Pretreated with DPI, ERK1/2 activity increased by 57% compared with the vehicle group (*P* < 0.01) and 50% compared with the IL-1*β* group (*P* < 0.01). ERK1/2 activity was stronger than that of the NAC group (*P* < 0.01), while p-ERK1/2 activity increased by 68% compared with the vehicle group (*P* < 0.01) but decreased by 50% compared with the IL-1*β* group (*P* < 0.001). The activation of p-ERK1/2 was weaker than that of the NAC group (*P* < 0.01) (Figures 6(f) and 6(g)).

After treatment with IL-1*β*, there was no significant difference in JNK1/2 activity between the vehicle group and the IL-1*β* group (*P* > 0.05), but p-JNK1/2 activity increased by 300% compared with the vehicle group (*P* < 0.001). Pretreated with GW1929, there was no significant difference in JNK1/2 activity between the vehicle group and the GW1929 group (*P* > 0.05) or the IL-1*β* group and the GW1929 group (*P* > 0.05) or the NAC group and the GW1929 group (*P* > 0.05), while p-ERK1/2 activity increased by 260% compared with the vehicle group (*P* < 0.01), but there was no significant difference in p-JNK1/2 activity between the IL-1*β* group and the GW1929 group (*P* > 0.05) or the NAC group and the GW1929 group (*P* > 0.05). Pretreated with DPI, there was no significant difference in JNK1/2 activity between the vehicle group and the DPI group (*P* > 0.05) or the IL-1*β* group and the DPI group (*P* > 0.05) or the NAC group and the DPI group (*P* > 0.05), while p-ERK1/2 activity increased by 240% compared with the vehicle group (*P* < 0.01), but there was no significant difference in p-JNK1/2 activity between the IL-1*β* group and the DPI group (*P* > 0.05) or the NAC group and the DPI group (*P* > 0.05) (Figures 6(h) and 6(i)).

## 4. Discussion

OA is characterized by gradual degeneration of articular cartilage, new bone formation, and synovial hyperplasia, which may eventually lead to pain, joint dysfunction, and disability [17, 18]. Clinically, pain and loss of joint function are the main problems that hamper the life of OA patients [19, 20]. So far, treatment options have been limited. At present, there is a lot of evidence that chondrocyte apoptosis is related to the characteristic cartilage degeneration of OA, although the mechanism involved has not been fully elucidated [21, 22].

The NOX family is mainly through the activation of ROS to complete the physiological and pathological functions. NOX of phagocytes has no activity in resting cells. ROS is activated when stimulated by pathogenic microorganisms, inflammatory mediators, and external factors, which are related to host defense. Different from phagocytic NOX, nonphagocytic NOX maintains a certain activity under physiological conditions and produces intracellular and extracellular ROS [9]. ROS produced through this pathway does not play a major role in cell defense, but as a “signal molecule” and “gene expression switch,” it participates in cell differentiation, proliferation, apoptosis, and the regulation of intercellular signaling pathways [23]. When the stimulation of extracellular factors is received, NOX family proteins are overexpressed and excessive ROS is produced, which is closely related to the occurrence and development of human diseases.

ROS is a kind of substance formed in aerobic metabolism and aerobic environment. It contains oxygen in molecular composition and has higher chemical activity than oxygen itself. The increase of ROS can cause serious oxidative damage to the main components of cells, such as DNA, protein, and lipid [24, 25]. Among them, DNA damage is the most common type, mainly including purine and pyrimidine bases, changes in DNA protein cross-linking, and breakage of oligonucleotide chains and base sites. Protein damage is the cleavage of peptide chains induced by ROS, which leads to direct oxidative modification of amino acid side chains [26]. Lipid peroxidation reduces the fluidity of biofilm and increases the permeability of the cell membrane, which led to apoptosis [10]. ROS can inhibit the synthesis of cartilage matrix proteoglycan, promote the degradation of proteoglycan and collagen, and affect the development of OA [27, 28]. In our experiment, the decrease of ROS fluorescence intensity was most obvious in the DPI group. There was no significant difference between the DPI group and the NAC group. Compared with the NAC group, the antioxidant stress effect of other groups was still lower than that of the NAC group (Figure 4(a)). It is confirmed that the activation of NOX in nonphagocytic cells can produce ROS and induce cell apoptosis (Figures 3 and 5). In terms of inhibiting apoptosis, the effect of each group was weaker than that of the NAC group, but all showed the effect of alleviating apoptosis in different degrees. Both activator and inhibitor can improve cell proliferation (Figure 2(b)).

ROS can directly attack mitochondria and cause oxidative damage. Mitochondrial damage in OA chondrocytes can increase the sensitivity of chondrocytes to inflammatory stimulation [29, 30]. During our experiment, the concentration of PGE_2_ in the GW1929 group, DPI group, and PD98059 group was lower than that in the NAC group, indicating that the GW1929 group, DPI group, or PD98059 group was stronger than the NAC group in anti-inflammatory effect. Although there was no significant difference between the SB203580 group and the NAC group or the SP600125 group and the NAC group, the concentration of PGE_2_ of the SB203580 group and the SP600125 group was lower than that of the IL-1*β* group (Figure 4(b)). These results indicate that PPAR*γ*, NOX2, and MAPK signaling pathways play a regulatory role in the production of PGE_2_. In downregulation of COX-2, the effect of the PD98059 group was weaker than that of the NAC group. There was no significant difference between the SB203580 group and the NAC group. But the expression of COX-2 in the SB203580 group and the PD98059 group was lower than that in the IL-1*β* group. The SP600125 group, the GW1929 group, and the DPI group had better effect on the downregulation of COX-2 than that of the NAC group (Figure 4(c)). It is speculated that PPAR*γ* and NOX2 had direct regulation on COX-2 as well as P38MAPK and JNK.

ROS is involved in signal transduction and is closely related to cell development and fate. ROS is the basis of the physiological function of the body. It regulates many kinds of signal transduction, protein structure, transcription factors, and genes through direct reaction and regulates their functions [31]. As a signal molecule, ROS promotes cell proliferation and differentiation by regulating the ERK1/2 pathway, while moderately increased ROS can induce apoptosis. So far, there are many ways to induce apoptosis by ROS. For example, ROS can induce JNK activation and induce apoptosis [11].

MAPKs are the key components of intracellular signaling pathways, which are related to cell proliferation, differentiation, apoptosis, cytokine response, and MMP expression [9, 32]. Studies have shown that MAPKs are widely involved in the signal transduction of articular cartilage degeneration, including ERK1/2, p38MAPK, and JNK. They play a negative role in the synthesis of the cartilage matrix [33]. In all MAPK signal transduction pathways, ERK1/2 is phosphorylated and activated by MAPK kinase 1/2 (MEK1/2). ERK1/2 is involved in many physiological and pathological processes, such as cell proliferation, differentiation, apoptosis, and cell function synchronization [34]. The P38MAPK signal transduction pathway is closely related to the maintenance and differentiation of chondrocyte phenotype, hypertrophy and calcification of chondrocytes, and apoptosis of chondrocytes, which may play a pivotal role in the destruction of OA chondrocytes [35]. The JNK pathway is mainly activated by extracellular stress, cytokines, and hypoxia and plays a proapoptotic role in cells [36].

MAPK signaling pathways play a key role in the signal transduction of OA cartilage injury, which receives extracellular stimulation to transfer into the nucleus, regulate transcription factors and their downstream genes, and finally react at the cellular level. Inhibition of MAPK signaling pathways can reduce lipid peroxidation of inflammatory cells, regulate apoptosis-related genes, and eventually lead to inhibition of cell proliferation and apoptosis, thus protecting articular cartilage [37]. P38MAPK inhibitors can be used to protect articular cartilage, and SB203580 is the most widely used. In the OA rat model, SB203580 can significantly slow down the process of cartilage degeneration, relieve pain, prevent the degradation of the extracellular matrix, and inhibit the expression of inflammatory factors such as COX-2, PGE_2_, and iNOS. In addition, SB203580 can inhibit the process of chondrocyte hypertrophy, reduce the synthesis of collagen X, and delay the aging process of chondrocytes [38, 39].

PPAR has three isomeric forms, PPAR*α*/*β*/*γ*, which are widely distributed in the human body. PPAR*γ* is the most deeply studied. Early studies mainly focused on the lipid metabolism regulation of PPAR*γ*, but recent studies have shown that PPAR*γ* also plays a role in the regulation of inflammatory response, matrix decomposition, and synthesis [40, 41]. It can significantly inhibit the expression of inflammatory cytokines and metalloproteins in various tissues and cells. It also negatively regulated the expression of AP-1, NF-*κ*B, and other inflammatory response genes [7, 42]. PPAR*γ* is present in all major cells of human joints, including chondrocytes. Recent studies have found that PPAR*γ* expression in OA patients or animal models is significantly reduced [43]. Downregulation of PPAR*γ* in chondrocytes will lead to structural and functional changes, such as decreased synthesis and secretion of the matrix of chondrocytes required, increased synthesis and secretion of matrix metalloproteinases, and increased release of inflammatory factors and cytokines. This effect is consistent with the changes of chondrocytes during OA [8]. Based on our results, we confirmed that the downregulation of PPAR*γ* was involved in the pathogenesis of OA. IL-1*β* induced oxidative stress, increasing chondrocyte apoptosis rates and decreasing PPAR*γ* protein activity, which consisted of the pathophysiology of OA. GW1929, as an agonist, can directly increase PPAR*γ* activity and thus inhibit oxidative stress. DPI can reduce ROS production by inhibiting NOX2 activity, which also inhibits oxidative stress (Figure 6). We inferred from our western blot that PPAR*γ* was not the nuclear transcription factor that directly activated NOX2 expression but may regulate NOX2 indirectly by activating other downstream proteins. The detailed mechanism underlying needs to be further confirmed. PPAR*γ* activation could downregulate the p38MAPK signaling pathway and inhibit NOX2. PPAR*γ* had no direct effect on ERK1/2, but NOX2 could regulate the phosphorylation of ERK1/2. Both PPAR*γ* and NOX2 had no effect on JNK.

## 5. Conclusion

IL-1*β* can induce the expression of COX-2 and PGE_2_ in chondrocytes through the NOX2/ROS/p38MAPK signaling pathway, and PPAR*γ* expression is downregulated during this period. Activation of PPAR*γ* can significantly inhibit the expression of COX-2 and PGE_2_ in chondrocytes induced by IL-1*β* and resist the injury of chondrocytes induced by IL-1*β*, so as to alleviate the pathogenesis of OA.

## Figures and Tables

**Table 1:** Primer sequences for qRT-PCR.

Gene	Forward (5′ → 3′)	Reverse (5′ → 3′)
COX-2	TGGCTTCGGGAGCACAAC	CAGCGGATGCCAGTGATAGAG
GAPDH	GGAGTCTACTGGCGTCTTCAC	ATGAGCCCTTCCACGATGC

## Data Availability

Data is available on request.
